# Intramural Gastric Abscess Misdiagnosed as Advanced Gastric Cancer

**DOI:** 10.34172/aim.2023.79

**Published:** 2023-09-01

**Authors:** Na-Ri Lee, Eun-Kee Song, So-Yeon Jeon

**Affiliations:** ^1^Division of Oncology and Hematology, Department of Internal Medicine, Jeonbuk National University Hospital-Jeonbuk National University Medical School, Jeonju, Republic of Korea; ^2^Research Institute of Clinical Medicine of Jeonbuk National University-Biomedical Research Institute of Jeonbuk National University Hospital, Jeonju, Republic of Korea

**Keywords:** Case report, Gastric cancer, Gastric wall abscess, Surgery

## Abstract

An intramural gastric abscess is a rare condition often mistaken for other medical diseases such as gastric cancer and neoplasms. We present a case of a patient initially believed to have pancreatic cancer based on his computed tomography scan. The clinical diagnosis of locally advanced gastric cancer was made on subsequent magnetic resonance cholangiography and endoscopic ultrasound (EUS). However, several EUS-guided biopsies did not reveal malignant cells. A partial gastrectomy was performed for diagnostic and therapeutic purposes. The specimen showed only inflammatory cells, without any malignant cells. The final diagnosis was gastric wall abscess (GWA) that infiltrated and adhered to the adjacent tissues. This case reminds that physicians should include GWA as a differential diagnosis in the suspicion of gastric cancer. Although GWA is rare, it is often forgotten when focusing on the possibility of fatal cancer.

## Introduction

 An intramural gastric abscess is a rare condition that may be difficult to diagnose based on imaging alone. Gastric wall abscesses (GWAs) are often misdiagnosed as stomach cancers.^1‒3^ GWA can be easily diagnosed with a secreting mass on endoscopy. However, in the absence of such discharge, diagnosis may be difficult, even after various imaging tests. Even if the discharge is present, its diagnosis may be difficult if other imaging studies consistently suggest malignancy. We report a case of GWA initially considered to be a pancreatic tail cancer directly invading the gastric wall, based on initial radiologic imaging.

## Case Report

 A 60-year-old man presented to our institution with abdominal pain, characterized as stabbing in nature, radiating from the epigastrium to the back. Although the patient did not recall his previous weight, he allegedly had significant weight loss.

 His abdominal pain began several weeks prior to admission. Abdominopelvic computed tomography (AP-CT) performed at the previous hospital indicated suspected pancreatic cancer. The patient was transferred to our hospital for diagnosis and treatment.

 The patient was diabetic and hypertensive. Five years prior, he had undergone extracorporeal shockwave lithotripsy for pancreatic duct stones. He was a regular alcoholic beverage drinker, consuming one or two cups of kaoliang 20 times a month for 30 years. His father died of gastric cancer several years ago.

 On physical examination, he had a body temperature of 36.9 °C, resting respiratory rate of 20 breaths/min, heart rate of 78 bpm, and blood pressure of 140/89 mm Hg. No abdominal or rebound tenderness was found.

 Laboratory examination revealed the following results: white blood cell count of 8390/mm^3^ (reference range [RR]: 3.5‒10.5 × 10^3^/mm^3^); hemoglobin of 12.0 g/dL; hematocrit of 34.5%; and platelet count of 267 × 10^3^/µL. His erythrocyte sedimentation rate was 98 mm/h (RR, < 9 mm/h), and his high-sensitivity C-reactive protein level was 130.47 mg/L (RR, < 5 mg/L). His CA 19‒9 level was 4.15 U/mL (RR, < 37.0 U/mL).

**Figure 1:**
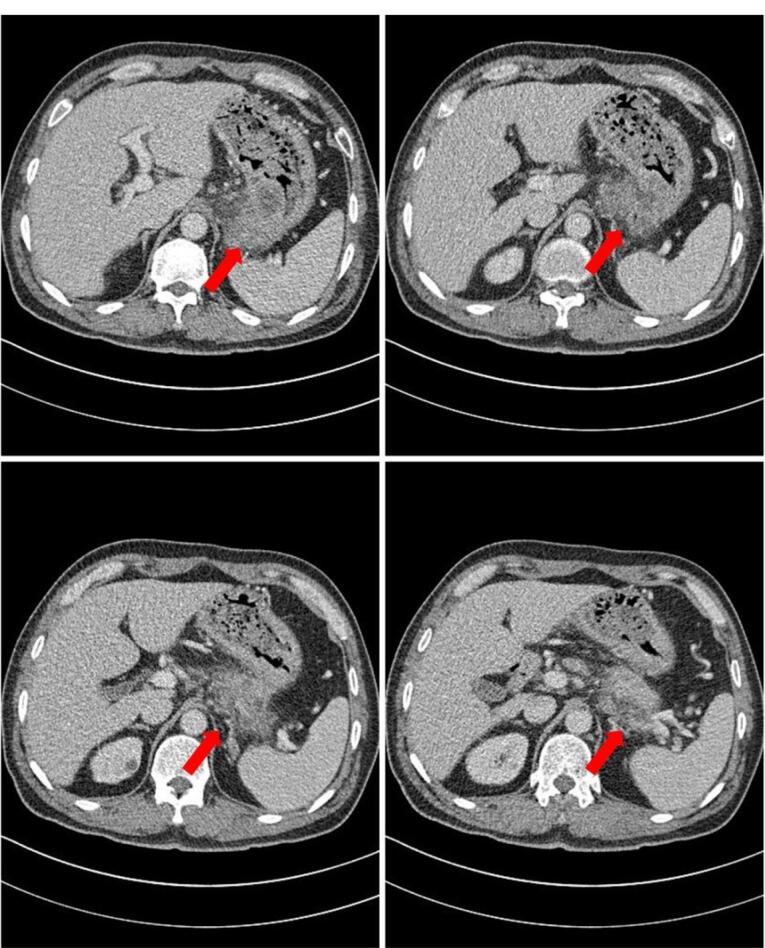
**Abdominopelvic computed tomography (AP-CT) Performed at the Local Clinic.** Pancreatic mass measuring approximately 7 × 4.5 cm and directly invading the gastric wall was observed, leading to a high suspicion of cancer

**Figure 2:**
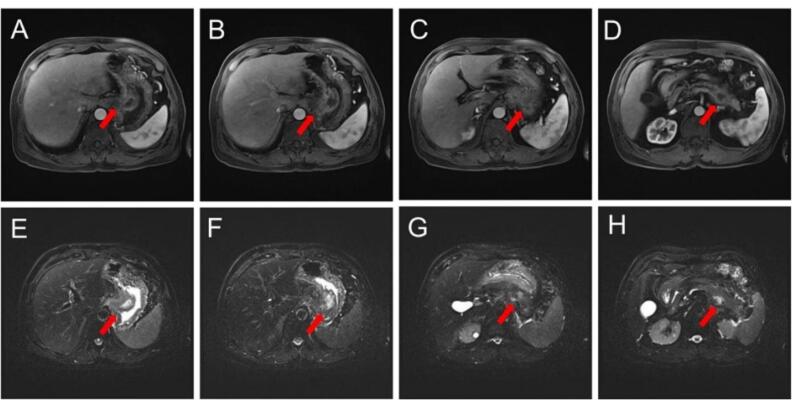
**Magnetic Resonance Cholangiopancreatography (MRCP) With Enhancement**. **A-D. **T1-weighted images. **E-H. **T2-weighted images. MRCP shows irregular wall thickening along the lesser curvature of the upper body of the stomach. The mass abutted the pancreas; however, the mass was not considered to originate from pancreatic ductal cancer

 AP-CT, performed in another hospital, revealed a pancreatic mass, measuring approximately 7 × 4.5 cm, that directly invaded the gastric wall. The mass was suspected to be a pancreatic malignancy (Figure 1). Magnetic resonance cholangiopancreatography (MRCP) was performed to localize the mass. MRCP showed irregular wall thickening of more than 5 cm in length along the lesser curvature of the stomach. The mucosal layer was almost maintained, and the wall thickening inside was observed as low density (Figure 2). Cystic or necrotic portions had invaded the splenic vessels behind the pancreas. The main mass abutted the pancreas; however, the pancreatic duct appeared intact, possibly indicating that the tumor mass did not originate from the pancreatic duct. Numerous enlarged lymph nodes were observed in the periportal and para-aortic areas; however, their size was too small for characterization. MRCP concluded that the malignant-appearing gastric mass had a retropancreatic extension.

**Figure 3:**
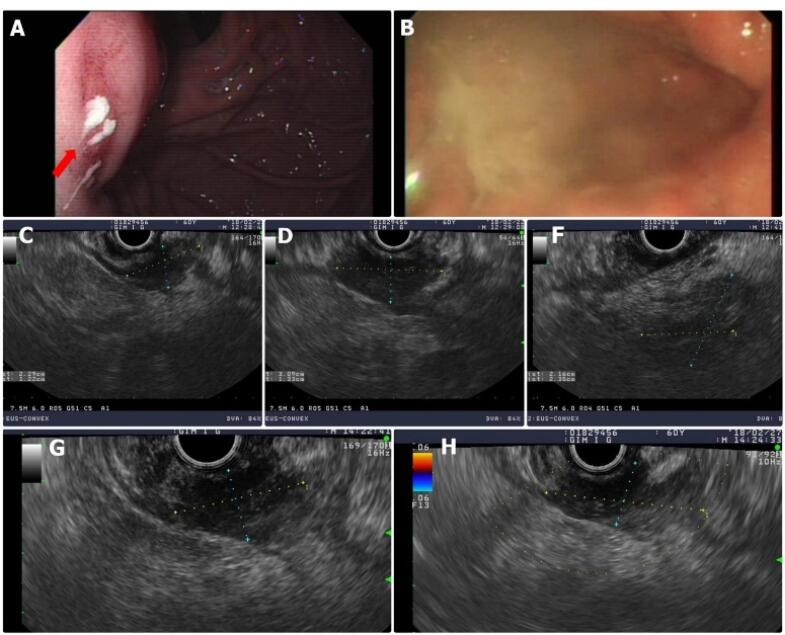
**Endoscopic ultrasound (EUS) Showing an Approximately 3 cm-Sized Mass in the Gastric Fundal Wall. A-B.** After EUS-guided fine needle aspiration, a whitish discharge oozed from the biopsy site. **C-G.** A mixed echoic mass measuring larger bigger than 3 cm with an irregular margin was noted. Gastric cancer was suggested based on EUS. Other possible diagnoses included retroperitoneal cancer, pancreatic cancer, and inflammatory mass of the stomach and retroperitoneum

**Figure 4:**
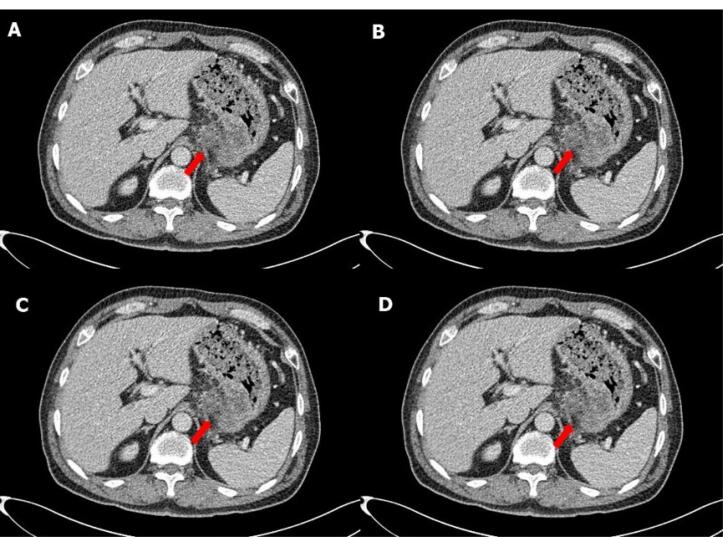
Follow-up Abdominopelvic Computed Tomography Showing a Decrease in Mass Volume by < 2 cm and Improvement in Retropancreatic Wall Extension

 Upper esophagogastroduodenoscopy (EGD) revealed erythematous bulging mucosa at the gastric fundus, oozing with whitish pus-like fluid (Figure 3). The endoscopist suggested the presence of gastric cancer or an inflammatory mass. We performed endoscopic ultrasound (EUS)-guided fine needle aspiration (FNA) and core needle biopsies. On EUS, we observed a gastric fundal wall mass measuring approximately 3 cm, with coarse echogenicity (Figure 3). Peripancreatic and retropancreatic masses > 3 cm in size were also observed, showing mixed echogenicity with irregular margins. Based on EUS findings, gastric cancer with retroperitoneum, splenic vein, and pancreatic invasion, with abscess formation in the gastric wall, was suspected. At this point, the differential diagnoses included retroperitoneal cancer with stomach invasion, pancreatic cancer with stomach invasion, and inflammatory masses of the stomach and retroperitoneum, in order of decreasing probability. Only acute suppurative inflammatory cells were observed on both FNA cytology and biopsies of the pancreas and stomach. A repeat EUS was performed, and FNA cytology and biopsies were consistent with an abscess in both the gastric wall and the pancreas. Because cancer could not be completely ruled out, a laparoscopic biopsy was performed. Preoperative AP-CT revealed that the mass volume had decreased to less than 2 cm, and the retropancreatic extension had markedly improved (Figure 4). Intraoperatively, adhesions were observed between the lower curvature of the stomach and the tail of the pancreas. There was no palpable hard mass suggesting malignancy. The adhesions were severe, so dissection was impossible, and we switched from laparoscopic to open surgery. The midbody of the stomach was removed by wedge resection, and the tail of the pancreas that adhered to the stomach was removed. All the specimens showed chronic inflammation without any malignant cells. The final diagnosis was GWA with pancreatic and retroperitoneal infiltrations.

 No antibiotics were administered because there were no clinical signs suggesting infection at the time of admission. However, prophylactic antibiotic (ceftezole 1 g every 12 h) was initiated preoperatively. The patient recovered well, without any signs of inflammation, and was discharged. On his outpatient follow-up visit, the patient remains in good health.

## Discussion

 GWA is a rare variant of suppurative gastritis. The diagnosis of GWA is made with the presence of an abscess with suppurative pus discharge. However, this finding is also present in other medical conditions, such as several types of subepithelial gastric neoplasms, lipomas, and hematomas. When an abscess directly invades adjacent organs, it can be confused with gastric cancer, gastrointestinal stromal tumors (GISTs), and neuroendocrine neoplasms.^2^ Therefore, early diagnosis of GWA is difficult, and as a result, its mortality rate is high.^4^ GWA diagnosis depends on a series of imaging examinations. EGD, CT, and EUS are crucial for distinguishing between gastric cancer and abscess.^1^ In the present case, the mass was misdiagnosed as pancreatic cancer on the initial AP-CT. However, MRCP suggested a diagnosis of gastric cancer with pancreatic and retroperitoneal infiltrations. On EUS, masses larger than 3 cm were observed in the gastric wall, pancreas, and retroperitoneum. Therefore, EUS findings suggested gastric cancer accompanied by gastric abscess and retroperitoneal invasion. GWA generally shows a thickened wall with a localized hypoechoic mass, which has heterogeneous echogenicity, mainly fluid echo and few gas or foreign body echoes in the muscular or submucosal layer on EUS.^4^ Serial MRCP and EUS examinations helped change our patient’s diagnosis from pancreatic cancer to gastric cancer, but did not raise the suspicion of GWA. Even though pus secretion, the most common diagnostic finding, was seen on EGD, it was thought to be a malignant tumor accompanied by abscesses. This is because there have been case reports of GWA associated with GIST or gastric cancer.^5‒7^ Since we could not obtain any malignant tissues, we decided to perform surgical resection for pathological confirmation and treatment. The radiologist interpreted the preoperative AP-CT as fluid collection accompanied by pancreatitis because the lesion was reduced to less than 2 cm and showed improvement. However, since malignancy was highly suspected, we ignored the possibility of benign disease.

 The mortality rate of GWA has decreased from 92% to 33%,^8^ due to advances in surgical resection and antibiotic treatment. The treatment success rate is higher in patients treated with surgical resection plus medical antibiotic therapy than those treated with only medical antibiotics. Surgical resection and antibiotic therapy are the mainstream treatments; however, surgical approaches probably result from the fact that GWA is difficult to diagnose preoperatively and is often misdiagnosed as a more common surgical problem, such as gastric cancer or stromal tumors.^8‒11^ Recently, therapeutic endoscopic interventions such as endoscopic and percutaneous drainage with antibiotic therapy have been attempted to replace surgery.^2,12^ However, when the disease is recurrent or complicated by stromal tumors, surgical treatment is needed.^2^ In the present case, we believe that the patient would eventually have to undergo surgery because GWA was complicated by infiltrating adjacent organs. Surgery allows accurate diagnosis and treatment at the same time and effectively lowers the mortality rate; therefore, surgery is still the mainstream treatment.

## Conclusion

 GWA is a rare condition which is sometimes difficult to diagnose radiologically. Most often, GWA is mistaken for gastric cancer. We report a case of a gastric abscess mimicking pancreatic cancer on initial radiological imaging. A series of imaging studies have suggested that the diagnosis is locally advanced gastric cancer. Even when pus discharge, the most diagnostic feature of GWA, was seen on EGD, concerns about malignant tumors continued to be intensively expressed. GWA was diagnosed and treated with open surgery followed by antibiotics. This case reminds physicians not to ignore or overlook the possibility of GWA owing to its rarity, even though it has typical clinical features, while focusing on the deadly cancer disease.
